# The Magnitude of Salt Intake Behaviors and Its Predictors among Saqez Urban Population of Kurdistan District in Iran: A Cross-Sectional Study

**DOI:** 10.1155/2022/8439517

**Published:** 2022-02-14

**Authors:** Kolsom Khoram, Mohammad Asghari-Jafarabadi, Mehrangiz Ebrahimi-Mamagani, Behjat Shokrvash, Maral Hariri-Akbari

**Affiliations:** ^1^Department of Health Education and Promotion, Faculty of Health, Tabriz University of Medical Sciences, Tabriz, Iran; ^2^Road Traffic Injury Research Center, Tabriz University of Medical Sciences, Tabriz, Iran; ^3^Department of Statistics and Epidemiology, Faculty of Health, Tabriz University of Medical Sciences, Tabriz, Iran; ^4^Social Determinant of Health Research Center, Tabriz University of Medical Sciences, Tabriz, Iran; ^5^Department of Biochemistry & Diet Therapy, Faculty of Nutrition & Food Sciences, Tabriz University of Medical Sciences, Tabriz, Iran; ^6^Medical Education Research Center, Health Management and Safety Promotion Research Institute, Tabriz University of Medical Sciences, Tabriz, Iran; ^7^Department of Foreign Languages, Faculty of Education, Yildiz Technical University (YTU), Istanbul, Turkey

## Abstract

**Background:**

Controlling and reducing salt intake are one of the solutions to overcome hypertension. This study aimed at determining the predictors related to salt control methods in Saqez urban population.

**Methods and Materials:**

In the present cross-sectional study, the sample population was randomly selected through cluster sampling. Data collection was performed using valid questionnaires, demographic, family economic status, knowledge, perception, intention, salt intake behaviors, and salt control methods, along with measuring body mass index (BMI) and hypertension levels. Descriptive, analytical statistical methods and multivariate logistic regression analysis were used to determine the predictors of desirable salt control methods. The variables of sex, age, family economic status, knowledge, perception, perceived social support, self-efficacy, and intention were analyzed as independent variables. Data analysis was performed using SPSS software version 24 at a significance level of 0.05.

**Results:**

Out of 766 participants, 73% were women, with mean (M) 32.83, standard deviation (SD) 9.52 years, and 77.2% were married. There were significant sex differences in employment (*P*=0.01) and economic status (*P*=0.016). The M (SD) of blood pressure (systolic/diastolic) was 110.65 (0.0212) (*P*=0.441). The salt intake control methods between men and women did not show significant differences (*P*=0.368). Among totally 88.5%, 87.7% men and 88.9% women followed desirable behaviors. The predictors that determine the adoption of salt control methods were sex (man) (OR = 0.71, 95% CI (0.38–1.29)), age (OR = 1.02, 95% CI = (0.99–1.05)), SES/FAS (medium, high level) (OR = 1.37, 95% CI = (0.754–2.47); OR = 0.46, 95% CI = (0.047–4.55)), blood pressure (OR = 1.33, 95% CI = (0.16–11.23)), knowledge (have) (OR = 1.01, 95% CI = (0.39–1.63)), intent to reduce salt (OR = 1.047, 95% CI = (1.03–1.06)), perceived salt reduction importance (OR = 1.02, 95% CI = (1.01–1.04)), perceived emotional support (health staff) (OR = 1.02, 95% CI = (1.01–1.04)), media (OR = 1.01, 95% CI = 0.99–1.02), perceived practical support (spouse) (OR = 1.02, 95% CI = 0.99–1.04)), and perceived self-efficacy (OR = 1.01, 95% CI = (0.99–1.03)).

**Conclusion:**

The support of health staff and spouse seems to be effective in controlling the salt intake behaviors of healthy individuals. In parallel with the development and change of people's lifestyles, new approaches (legal and services) for salt control based on the support of media and social media were expected.

## 1. Introduction

“Hypertension” is one of the main risk factors for cardiovascular disease [1–3]. About one billion people worldwide have hypertension, and nearly eight million die of hypertension every year [4–6].

The relationship of salt intake with hypertension [1], heart disease, gastric cancer, osteoporosis [4, 5, 7], kidney disease, and proteinuria is known [8]. According to experts, daily consumption of less than 5 grams of salt (2 grams of sodium) in healthy people and 4 grams of salt (1.5 grams of sodium) in patients with hypertension creates no danger [1, 9–11]. Research findings from around the world showed that not only healthy people but even patients do not follow the advice of experts. In general, salt intake is high (9–12 gr) among not only healthy populations, but also overweight patients [9, 12, 13]. The 30% reduction of salt (sodium) intake by 2025 is one of the both national and international goals of the World Health Organization (WHO) [7, 14, 15].

Adding salt during preparing, cooking, and eating food or consuming foods high in salt result in excessive salt intake [16–20]. The pattern of salt intake in industrial societies is different from traditional societies [21]. In most traditional societies, more than three-quarters of the salt received is through homemade food items and during cooking. In contrast, salt intake in industrialized countries is through the consumption of processed and preprepared foods [21].The salt intake control requires the adoption of appropriate strategies harmonized with the salt intake manner of populations. Numerous solutions have been proposed to control and reduce the salt intake and its adverse consequences [1, 22–26]. Applying appropriate or community-based strategies [13, 25], modifying the salt intake pattern through enhancing awareness [25] and empowering populations were introduced as a cost-effective way to achieve healthy life [1].

Given the high prevalence of hypertension in Iran and the national commitment to salt intake reduction by 2025 [15], a comprehensive review of the salt intake model is necessary to develop and adopt appropriate solutions for excessive salt intake. According to the available evidence, the salt intake in Iran is 2–3 times more than the normal allowance [27–31]. Lack of knowledge about the recommended amount of salt intake [16, 31] is one of the predictors of adding excessive salt during preparing and eating food among healthy people as well as patients with hypertension [16, 29–31].

The general aim of this study was to determine the prevalence of salt control methods and its predictors, alongside to determine salt intake behaviors during cooking and or eating among Saqez urban population of Kurdistan district of Iran. Research question: how did the salt intake control behaviors during cooking and or eating, function among Saqez urban population of Kurdistan district of Iran?

## 2. Methods and Materials

### 2.1. Study Population

In the present cross-sectional study, the sample population was chosen from Saqez urban population covered by health centers in Saqez city.

### 2.2. Eligibility Criteria

It includes head of the family, women, and/or men 18 and above, without the history of hypertension, content to participate in the study, and having informed consent.

### 2.3. Sample Size Determination

The sample size of the study was estimated based on the Morgan table with 95% confidence and tolerable error of 5%, and including the large statistical population was estimated at 384. According to the cluster sampling method, the number of samples was estimated to be twice the volume, i.e., 766.

### 2.4. Sampling Technique and Procedures

According to the latest statistics, a total of 11 comprehensive urban health centers covered a population of 125,383 people. The sample population was randomly selected from the list of households covered by each health center (as a selected cluster) based on the inclusion criteria (no known disease, able to respond, and agree to participate in the study). After coordination with the officials of the health centers, the samples should come to the center through a formal invitation to participate in the research on a certain day. On data collection day, while stating the purpose of the research, the reason for their selection, how to cooperate, and how to complete the questionnaires, the confidentiality of participants' personal information was clarified to the participants, and it was also included in the first sheet of the questionnaire. The research was conducted after obtaining permission from the research ethics committee [IR.TBZMED.REC.1397] of Tabriz University of Medical Sciences and in coordination with the Deputy of Health of Saqez city.

### 2.5. Data Collection and Measurements

Data were collected using questionnaires with anthropometric tools.

#### 2.5.1. Questionnaires

The demographic questionnaire included sex (man, woman), age (year), level of education (year), employment, and family size of the participants. The family economic status was determined according to the valid family affluence scale (FAS) [32]. The possible range was 0–48; the score below 17 was grouped as lower class, 18–28 as middle class, and 29 and above as upper class.

The knowledge questionnaire consisted of five items. Four questions were regarding the amount of salt equivalent to a teaspoon (tsp), salt allowance for healthy people, impacts of high salt intake, and salt intake-related diseases [33–35]. The last special question is to assess the knowledge about salty and local salted foods (24 items) in the community. A list of the salty foods was provided as different kinds of bread, salt-soaked rice, roasted rice, salt-soaked bread, salt-smoked fish, salted-dried vegetables, pickles, pastes, cheese, dried-salted cheese, dried yoghurt, preprepared foods, processed foods, sausage, potato chips, salted nuts, crackers, noodles, canned foods, and food dressing [36].

Likert scale was used from very low, low, medium, and high to very high in a range of 1 to 5, respectively. Responses were scored based on true/know or not true/do not know, recoded as desirable knowledge or undesirable knowledge. For analysis, knowledge was calculated both generally and specifically. The range of total awareness score was 28 (4.24). Awareness scores were normalized between 0 and 100.

Participants' perceptions included perceptions of the importance of salt control, perceptions of social support from different sources, and perceptions of their ability to control salt intake effectively or self-efficacy [32]. Perception of the importance of reducing salt intake through self-controlling was assessed using five questions [33]. Questions included are as follows: (1) perceptions of the importance of salt reduction in food for personal health, (2) The importance of low salt intake among healthy people, (3) the importance of low salt intake among elderly, pregnant women, and patients, (4) the importance of low salt intake to maintain heart health and, (5) the importance of reducing and controlling the amount of salt use in different foods. Answers were scored on the Likert scale as very low, low, medium, high, and very high, and then were normalized on a scale of 0–100. A high score meant high perception. Questionnaire measuring perception of social support [32] included questions about perception of emotional-informational and practical support from the media, health personnel, spouse, family, and friends. Questions evaluating perception of social support included 20 items: emotional-informational support from spouse and family (three items); practical support from spouse and family (five items); friends (4 items), media (4 items), and health personnel (4 items) to control and reduce the amount of salt intake. The Likert point scale (very low, low, medium, high, and very high) was scored from 1 to 5 and normalized between 0 and 100, respectively.

Measuring the degree of self-efficacy or self-confidence in controlling and reducing salt intake by the individual and the family consisted of 8 questions taken from existing and other valid questionnaires [32]. To measure self-efficacy, questions included are as follows: I can prepare low-salt foods, even if it costs more money; I can encourage eating low-salt foods in the family, even if it is not tasty; I can encourage eating low-salt foods in the family, even if they do not like it; I can reduce the amount of salt in foods, even if the spouse does not like it; I can control the amount of salt intake in the family; I can use healthy and low-salt flavors and side foods; I can reduce the intake of high-salt foods. The intention or decision of the participants to adopt salt control behaviors was measured with 4 items: I intend to reduce my salt intake; I intend to reduce family salt intake; I intend to increase preparation of low-salt foods; I intend to purchase and use healthy seasonings and flavors instead of salt. The answers were scored according to the Likert scale (1 = strongly disagree, 2 = disagree, 3 = agree, and 4 = strongly agree) and then were normalized as 0–100 ranging.

#### 2.5.2. Salt Control Methods Questionnaire

To determine salt control methods, a list of healthy behaviors was developed according to the WHO recommendations [5, 35, 37]. Salt control methods included eliminating or reducing processed foods, not adding salt during cooking, not adding salt when eating, choosing and eating low-salt foods, using flavors or seasonings as salt substitutes, avoiding preprepared meals, avoiding eating out, and reading food labels. Responses were scored based on “doing” and “not doing” recoded as “desirable behavior = 1” and “undesirable behavior = 0.”

Evaluating salt intake behaviors was done specifically using two questions about adding salt during food preparation process and adding salt while eating. Measurement of salt intake was determined on the basis of a home quantitative scale, one teaspoon (tsp) (equivalent to 5 g) adapted to international standards [16, 36].

Frequency of daily intake of high-salt food items of the household was determined using a semiquantitative valid food frequency questionnaire [36].The questionnaire included 32 items of common high-salt food in the community, as mentioned above.

The questionnaire for illiterate people was completed by the interviewer. The research was conducted in Saqez area from Feb 2018 to Jun 2019.

#### 2.5.3. Anthropometric Tools

Body mass index (BMI) and blood pressure of participants were determined. To determine body mass index, height was measured using a special adult height gauge in centimeters and weight was measured in kilograms with a minimum of clothing by a weight gauge and waist circumference with a tape measure and documented. Calculation of participants' BMI was done by the WHO standard [38]. The results were classified into three groups: BMI < 18.5 kg/cm^2^ underweight; BMI = 18.5–24.9 normal weight; BMI = 25–29.9 kg/cm^2^ over weight.

Hypertension was evaluated by a health expert through measuring systolic and diastolic blood pressure using a mercury sphygmomanometer in millimeters of mercury under standard conditions and in a sitting position. Then, it was classified according to the valid criteria [39]. Systolic and diastolic blood pressure equal to or greater (≥) than 140/90 was defined as hypertension.

### 2.6. Statistical Methods

Descriptive and analytical statistical methods were used to analyze the data. Data related to qualitative variables were determined by frequency and percentage; and the quantitative variables were determined by M(SD). Chi square test and independent *t*-test were used to determine the differences in data distribution between participants. Multivariate logistic regression analysis was used to determine the predictors of salt control methods.

A list of common salt control behaviors were determined based on doing or not doing in the two groups as desirable and undesirable methods, and desirable methods were considered as an outcome variable which were entered into the model. The variables of sex, age, family economic status, knowledge, perception, perceived social support, self-efficacy, and intention were analyzed as independent variables. Data analysis was performed using SPSS software version 24 at a significance level of 0.05.

### 2.7. Ethical Considerations

The proposal and questionnaires were approved by the Ethics Committee of Tabriz University of Medical Sciences, code: P/406. All participants entered the study after signing the consent form. They had liberty to leave the study (mentioned on the first page of the questionnaire).

## 3. Results

### 3.1. Demographic Characteristics of Participants

A total of 766 adults, 73% women, and 77.2% married with a M(SD) age of 32.83 (9.52) participated in this study. Employment distribution (*P*=0.01) and family economic status (*P*=0.016) of participants showed significant differences (Table 1).

### 3.2. Anthropometric Indicators of Participants

M(SD) of weight was 83.72 (44.27), BMI was 26.74(4.04), and systolic/diastolic blood pressure was estimated as 110.65 (12.02). No significant difference was observed in terms of sex (*P*=0.441) (Table 1).

### 3.3. Knowledge of Participants

Knowledge of the amount of one teaspoon of salt equivalent to 5 grams (gr) in women and men showed significant differences 26.2 and 20.6, respectively (*P* < 0.001). Knowledge of the daily allowance of salt according to the recommendations of experts, women (47%) and men (53.1) showed somewhat significant differences (*P*=0.056) (Table 2).

Knowledge about the side effects of high salt intake or high-salt diet, 95.7% answered correctly. The answers did not show a significant difference based on sex without mentioning any disease (*P*=0.236). In total, 95.7% of the participants were aware of the adverse effects of a high-salt diet (excluding disease), about half of the participants (48.4%) being aware of hypertension and 71.5% of hypertension and one or more salt-related illnesses. Knowledge of the connection between hypertension and high salt intake was 2.87% among women and 9.81% among men.

M(SD) general knowledge of participants about the equivalent amount and scale recommended the amount of salt and salt-related diseases was 28.51 (22.43) without any significant difference (*P*=0.704). Nearly twenty percent (19.9) were aware of the amount of salt, related diseases, and high-salt foods (*P*=0.454). Except for the knowledge of the scale and its equivalent amount, and the recommended amount of salt (*P*=0.001) (0.056), salt-related diseases did not show significant differences by sex (Table 2).

### 3.4. Perception of Social Support, Self-Efficacy, and Intention of Participants

The perception of emotional-informational support as well as practical support from television (TV), radio, and magazines were significant (*P* < 0.001, *P*=0.008). Perception of the support of spouse, health personnel, and friends did not show a significant difference (*P*=0.504). The M(SD) score of perception of one's ability to control salt and high-salt foods was 64.98 (16.18) for women and 65.37 (17.83) for men (*P*=0.778) (Table 3). The M(SD) score of intention to control salt intake in men was 79.96 (19.38) and women 78.94 (19.28). Intention score did not show significant difference by sex (*P*=0.525) (Table 3).

### 3.5. Salt Intake Control Methods among Participants

The salt intake control methods between men and women did not show significant differences (*P*=0.368). Among totally 88.5%, 87.7% men and 88.9% women followed desirable behaviors.

The behavior of adding salt as much as one tsp or less when preparing food (*P*=0.201) and eating food (*P*=0.157) did not show a significant difference between women and men. In this study, 88.5% of the population controlled their salt intake and that of their families. No significant difference was observed in the behavior of men and women (*P*=0.368) (Table 3).

### 3.6. Predictors of Salt Control Methods among Participants

Multivariate logistic regression was used to determine the predictors of desirable salt intake control methods of Saqez families. The results showed sex (man) (OR = 0.71, 95% CI (0.38–1.29)); age (OR = 1.02, 95% CI = (0.99–1.05)); SES/FAS (medium, high level) (OR = 1.37, 95% CI = (0.754–2.47); OR = 0.46, 95% CI = (0.047–4.55)); blood pressure (OR = 1.33, 95% CI = (0.16–11.23)); knowledge (know) (OR = 1.01, 95% CI = (0.39–1.63)); intent to reduce salt (OR = 1.047, 95% CI = (1.03–1.06)); perceived salt reduction importance (OR = 1.02, 95% CI = (1.01–1.04)); perceived emotional support (health staff) (OR = 1.02, 95% CI = (1.01–1.04)); perceived practical support (spouse) (OR = 1.02, 95% CI = 0.99–1.04)); and self-efficacy (OR = 1.01, 95% CI = (0.99–1.03)) were predictors of adopting of salt control methods in the Saqez population(Table 4).

## 4. Discussion

The prevalence of salt control methods and its predictors in the population covered by Saqez health centers was investigated. The majority of the population followed salt control methods without sex differences. Sex (man), age, family economic status, knowledge, perception of the importance of salt control, emotional support from health personnel, practical support of the spouse, and self-efficacy were identified as predictors of the desirable salt control behaviors.

Despite the prevalence of adding salt behavior during cooking, the majority of the study population controlled their salt intake and that of their families. Salt control behaviors include a series of healthy behaviors related to removal and reduction of salt [33–35]. These behaviors include not adding salt when cooking or eating, choosing and eating low-salt foods, using healthy homemade condiments and salt substitutes, avoiding ready-to-eat foods, eliminating or reducing processed foods, not eating out, and reading food labels. Salt control is considered as a desirable healthy behavior.

The behavior of adding salt during cooking was assessed as undesirable. Behavior of adding salt while eating was less common among both women and men. More than half of the participants added more than one tsp salt during food preparation or cooking. These findings are inconsistent with previous findings [12, 13, 40]. In a study conducted among patients, the behavior of adding salt during cooking was more common among women while adding salt during eating was among men [16]. According to Anderson, in traditional societies, adding salt is more common than in nontraditional societies during cooking and eating [21]. Adding salt to food is done as a usual routine, just to make food tasty [21]. Controlling the behavior of adding salt requires the adoption of interventional methods in comprehensible ways.

In this study, salt intake control behaviors were observed almost equally among men and women. Although the perception of population was poor and did not show a significant difference in terms of sex, the specific knowledge about the amount of salt recommended by experts and the salt measurement scale was significantly different. Nearly half of the participants were aware of the recommended amount of salt. International findings showed that more than two-thirds of people in Australia, Germany, the United States, and South Africa were unaware of the amount of salt allowances and salt intake sources. In China, 34% of people and in the United States, 3% were aware of the recommended amount of salt and salt sources. Excessive salt intake differences between populations are likely due to low awareness [41]. This study comes to agreement with Land et al. who noted there was no significant association between knowledge, attitudes, and behaviors towards salt intake [42].

Salt control behaviors were more common among housewives with low economic status. As family economic status improved, salt control behaviors decreased. It is likely that higher economic status results in more easy access to purchasing ready-to-go food items from stores. Therefore, in modern and industrial societies, salt intake becomes out of one's control because of consuming high-salt food items, which requires legal and service approaches and interventions [21].

Intention is considered as one of the preconditions both for adopting and performing a behavior [43]. Those participants who intended to reduce salt intake had a better chance to adhere the optimal salt control behavior. Contrary to this finding, Newson et al., found that more than a third of the study population had no intention of salt reduction for the next six months. Actually most Germans and Australians were not interested in salt reduction despite their high salt intake. Given the different interests and intentions of populations in communities, specific approaches are needed to promote and adopt healthy behaviors [41].

The role of media and social media in promoting healthy behaviors has been identified as cost-effective [41, 44]. In the present study, the participant perception of support from different sources showed that the emotional-informational support from health personnel and practical support from the spouse equally predicted the salt control behaviors. However, the predictive role of media support for salt control behaviors was assessed as a weakness. The distribution of perception scores on emotional support was significantly different from the media in terms of sex. Numerous reasons can be given to describe the weak supportive role of the media in salt control among the population. One reason can be related to the nature of the mass media program. Media health programs are likely produced to be professional-level and not publicly perceived. Another reason may be due to the lack of interest or desire of the population to watch TV programs. International findings showed that most people are eager to gain knowledge, logic, and the reasons for low or no salt intake. In addition, 35% of the participants were interested in obtaining information from doctors and nutritionists. About 40% of the participants described TV and 18% the social media as a proper medium to obtain information about salt consumption [41].

### 4.1. Limitations

This research did not include questions regarding the preferred sources of obtaining information. Besides, vegetable and fruit intake of participants was not taken into the consideration. As a cross-sectional and questionnaire-based research, the study also suffers from some weaknesses in this regard.

## 5. Conclusion

The majority of the population followed salt control methods without sex differences. Perceived emotional and practical support from health staff and spouse was high, but it was low from media side.

Given the growing prevalence of noncommunicable diseases, especially hypertension in Iran [7], the supportive role of health professionals, especially the media, is essential in promoting healthy behaviors among high middle class population. The development of urbanization and the reflection of its adverse consequences on people's lifestyles make it essential to create and implement legal actions along with supervision and monitoring the salt control intake.

It is also beneficial to encourage adopting optimal personal behaviors and healthy choices through proper media. By identifying the media and social media of people's interest, appropriate content can be developed for the public and healthy patterns of behavior can be promoted with effective methods and strategies. On the one hand, it is necessary to manage salt control behaviors in individuals by relying on the power of health and nutrition experts and strengthening family support (spouse). More in depth future studies are needed to investigate the reasons for adding salt during food preparation and not adding salt during eating.

## Figures and Tables

**Table 1:** Participants' characteristics.

	Man	Woman	All	*P* value

Age (year)	28.33 (44.9)	66.32 (55.9)	83.32 (52.9)	0.432^a^

Occupation				0.001^b^
Employed	203 (98.5)	172 (30.8)	375 (49.1)	
Unemployed	3 (1.5)	386 (69.2)	389 (50.9)	

Education (year)				0.230^b^
12 and above	142 (68.9)	357 (64)	499 (65.3)	
Under 12	64 (31.1)	201 (36)	265 (34.7)	

FAS (item)^c^				0.016^b^
Low (0–17)	114( 23.7)	368 (76.3)	482 (63.3)	
Medium (18–28)	90 (33.3)	180 (66.7)	270 (35.5)	
High (29 and above)	2 (22.2)	7 (77.8)	9 (1.2)	

Weight (kgr)				0.001^a^
M(SD)^d^	81.87 (48.17)	69.51 (11.73)	72.83 (27.44)	

Waist (cm)				0.562^a^
M(SD)^d^	88.70 (15.29)	88.10 (11.31)	88.26 (12.49)	0.562

Waist category				
Low^e^	188 (91.3)	264 (47.3)	452 (59.2)	0.001^a^
High^f^	(8.7) 18	(52.7) 294	312 (40.8)	

BMI (weight^kgr^/height ^cm2^)				
M(SD)^d^	26.17 (3.85)	26.94 (4.10)	26.74 (4/04)	0.018^a^
Underweight < 18.5	8 (61.53)	5 (38.46)	13 (1.7)	0.025
18.5–24.9	71 (28.17)	181 (71.82)	252 (32.9)	
25–29.9	97 (26/35)	270 (73.36)	368 (48)	

Blood pressure (mmHg)				
M(SD)^d^	115.78 (12.28)	110.64 (43.99)	110/65(12.02)	0.441^a^

Systolic/diastolic pressure category *N*(%)				0.001^a^
Under 140/90	201 (97.6)	547 (98.0)	748 (97.9)	
140/90 and above	5 (2.4)	11 (2.0)	16 (2.1)	

^a^ *P* value based on *t*-test. ^b^*P*value based on chi square test by exact procedure. ^c^FAS, family affluence scale. ^d^M(SD), mean (standard deviation). ^e^Waist category for women, low <88 (cm), ^f^high ≥88 (cm) and above. ^g^mmHg, millimeters of mercury.

**Table 2:** Distribution of frequency and percent of participants' knowledge according to sex.

	Man, *N* = 206 (27%)	Woman, *N* = 558 (73%)	All, *N* = 766 (100%)	*P*value

Specific knowledge				
1 tsp equals 5 gr				0.001^d^
Know	51 (26.20)	113 (20.80)	164 (22.30)	

Recommended salt amount for healthy people				0.056^d^
Know	102 (53.10)	252 (47.)	345 (48.60)	

Impact of extra salt intake				0.236^d^
Know	185 (96.90)	503 (95.40)	692 (95.70)	

High salt foods				0.454
Know	42 (20.40)	110 (19.70)	152 (19.90)	

General knowledge (score)				0.704^e^
M(SD)^b^, min = 0, max = 100	28.03 (23.24)	28.72 (22.14)	28.51 (22.43)	

General knowledge				0.454^d^
Know	42 (20.40)	110 (19.70)	152 (19.90)	

Perceived importance of salt reduction				
M(SD)^b^	77.87 (18.86)	79.02 (21.85)	79.22 (19.29)	0.504

^a^1 tsp = 5 gr, one teaspoon equals 5 grams; ^b^M(SD), mean (standard deviation); ^d^*P* value based on chi square; ^e^p-value based on independent t-test.

**Table 3:** Distribution of frequency and percentage of participants' perception of social support, self-efficacy, and salt intake behavior according to sex.

	Man, M(SD)^a^	Woman, M(SD)^a^	All, M(SD)^a^	*P* value^b^

Perceived emotional support				
Health staff	66.07 (25.08)	67.59 (25.23)	67.18 (25.18)	0.464
Spouse	50.27 (25.56)	49.95 (25.99)	50.03 (25.86)	0.880
Media	39.96 (26.12)	47.56 (26.62)	45.51 (26.68)	<0.001
Friend	31.34 (27.51)	35.07 (25.52)	34.06 (26.11)	0.082

Perceived practical support				
Health staff	58.62 (22.89)	57.50 (25.08)	57.81 (24.50)	0.580
Spouse	51.75 (22.25)	49.39 (21.96)	50.03 (22.05)	0.197
Media	42.49 (25.32)	47.92(24.78)	46.45 (25.03)	0.008
Friend	35.16 (26.28)	38.75 (26.10)	37.78 (26.18)	0.095

Self-efficacy	65.37 (17.83)	64.98 (16.18)	65.09 (16.63)	0.778

Intent to reduce salt	79.96 (19.38)	78.94 (19.28)	79.22 (19.29)	0.525

Salt intake control methods	64.63 (12.40)	65.49 (13.27)	65.25 (13.04)	0.422

N (%)	178 (87.70)	437 (88.90)	665 (88.50)	0.368

Salt intake behavior				

Adding salt during cooking				0.201
Adding more than 1 tsp	117 (57.10)	333 (60.80)	450 (59.80)	
Adding 1 tsp or less^c^	88 (42.90)	215 (39.20)	303 (40.20)	

Adding salt during eating				0.157
Adding more than 1 tsp salt	20 (10.10)	70 (13.2)	90 (12.40)	
Adding 1 tsp or less^c^	178 (89.90)	460 (86.80)	638 (87.60)	

^a^M(SD), mean (standard deviation); ^b^*P* value based on *t*-test; ^c^1 tsp, one teaspoon.

**Table 4:** Result of multivariate logistic regression analysis for control salt methods as a dependent variable (optimal or desirable behaviors = 1).

	OR^a^	95% CI^b^ (lower–upper)	*P* value^c^

Sex			
Woman	Ref.^d^		
Man	0.71	0.38–1.29	0.263

Age (year)	1.019	0.99–1.05	0.236

SES^e^ (item)			
Low (0–17)	Ref.^d^		
Medium (18–28)	1.37	0.754–2.47	0.303
High (29 and above)	0.46	0.047–4.55	0.508

Blood pressure			
<140/90^f^			
≥140/90^g^	1.33	0.16–11.23	0.791

General knowledge			
Do not know	Ref.^d^		
Know	1.01	0.39–1.63	0.549

Intent to reduce salt intake (score)			
	1.047	1.03–1.06	<0.001

Perceived importance of salt reduction (score)			
	1.023	1.01–1.04	0.001

Perceived emotional support (score)			
Health staff	1.02	1.01–1.04	0.005
Spouse	0.98	0.96–1.10	0.012
Media	1.01	0.99–1.02	0.817
Friend	1.00	0.984–1.02	0.938

Perceived practical support (score)			
Health staff	0.99		
Spouse	1.02	0.99–1.04	0.064
Media	0.99	0.98–1.01	0.707
Friend	0.99	0.98–1.02	0.938

Self-efficacy (score)			
	1.01	0.99–1.03	0.338

Results: Hosmer and Lemeshow test: chi square (8) = 10.64; *P* value = 0.223; accuracy = 91.1%; ^a^OR, odds ratio; ^b^95% CI, 95% confidence interval; ^c^*P* value, ^d^referent group; ^e^SES, socioeconomic status based on family affluence items; ^f^<, under 140/90; ^g^≥, equal and above.

## Data Availability

The raw data which were used to support the findings of the study are available from the corresponding author, email: shokrvash@tbzmed.ac.ir, upon reasonable request.
